# Flame treatment of graphene oxides: cost-effective production of nanoporous graphene electrode for Lithium-ion batteries

**DOI:** 10.1038/srep17522

**Published:** 2015-12-10

**Authors:** Hao-Bo Jiang, Yong-Lai Zhang, Yi Zhang, Yan Liu, Xiu-Yan Fu, Yu-Qing Liu, Chun-Dong Wang, Hong-Bo Sun

**Affiliations:** 1 State Key Laboratory on Integrated Optoelectronics, College of Electronic Science and Engineering, Jilin University, 2699 Qianjin Street, Changchun 130012, China; 2 College of Physics, Jilin University, 119 Jiefang Road, Changchun, 130023, China; 3 College of Chemistry and Molecular Sciences, Wuhan University, Wuhan, 430072, China; 4 School of Optical and Electronic Information, Huazhong University of Science and Technology, Wuhan, 430072, China; 5 Key Laboratory of Bionic Engineering (Ministry of Education), Jilin University, Changchun 130022, China

## Abstract

A facile production of highly porous graphene foam by using flame treatment of graphene oxide (GO) is proposed. Highly porous architectures with randomly distributed micro-crack and micro-slit were produced due to the high temperature induced ruinous reduction and rapid expansion of GO. Synchronously, abundant oxygen-containing groups (OCGs) on GO sheets could be effectively removed after flame treatment, which renders significantly increased conductivity to the resultant flame reduced GO (FR-GO). The synergistic effect of micro/nanostructuring and the OCGs removal makes FR-GO a promising candidate for electrode materials. Compared with chemically reduced GO (CR-GO), FR-GO delivers much higher specific capacity. It gives us some hints that flame treatment of graphene-based material is a smart strategy for cost-effective production of anode materials for commercial application.

Lithium-ion batteries (LIBs) are considered as a big achievement in electrochemical energy conversion and storage for both daily necessities and industrial demands (such as cellular phones, laptop computers, electric vehicles)^1^. Development of high energy density and long cycle lifetime LIBs is in high demand, which depends on both the physical and chemical properties of electrode materials^2^,^3^. Up to now, various anode materials, ranging from metal oxides/sulfide (*e.g.*, SnO_2_^4^, TiO_2_^5^, Fe_2_O_3_^6^, Co_3_O_4_^7^, NiO^8^, MnO_2_^9^, MoO_3_^10^, WO_3_^11^ and MoS_2_^12^), nitrides^13^, polymers^14^, to lithium alloys (Si, Sn, Al, Sb)/multinary alloys, have been well investigated for the development of high-performance LIBs. Although most of the metal oxide and sulfides deliver much higher theoretical capacities than that of carbon (a theoretical specific capacity of 372 mAh/g)^15^, the low cycling stabilization and high manufacturing cost of such materials hinder the process for practical production and commercialization as anode materials. From this point of view, development of novel strategies that allow cost-effective and mass production of carbon material towards outstanding LIBs application is still a big challenge.

As a new family member of carbon materials, graphene, which is a single layer of two-dimensional (2D) honeycomb-structured carbon lattice, is promising to achieve outstanding electrochemical performance^16^,^17^ due to the high electrical conductivity^18^, high surface area (over 2600 m^2^g^−1^)^19^, flexibility^20^, mechanical strength^21^ and its potential in optoelectronic devices^22^. For development of high-performance graphene anodes, the formation of highly porous structure is essential, since a large contact area between the electrode and electrolyte can effectively shorten the transportation path length of Li ions and improve the electrochemical reaction kinetic, which results in good cycling performance as well as rating performance. In this regard, great efforts have been devoted to the preparation of nanostructured graphene in order to achieve remarkably enhanced surface area. As typical examples, X. Zhao *et al.*, prepared a flexible holey graphene paper by introducing in-plane pores into graphene sheets *via* a mechanical cavitation-chemical oxidation approach^23^. Yin *et al.*, assembled GO sheets into hierarchical structures by a “breath figure” method, leading to a high performance anode with reversible capacity up to 1600 mAh/g. Recently, it is also found that optical approach for preparation of porous graphene is also very powerful^24^. By using photoflash and laser, N. Koratkar *et al.*, achieved a reduced free-standing GO paper as high-rate capable anodes for LIBs^25^. The photothermally reduced GO anodes exhibit a unique “open-pore” structure due to the drastic expansion, which enables access of lithium ions to the underlying graphene sheets and facilitates efficient intercalation kinetics even under ultrafast charge/discharge rates^26^. Though big progress has been achieved in preparation of highly porous graphene matrix, the complex experimental process, high-cost and low-yields make these methods unsuitable for practical mass-production, let alone further commercialization.

In this work, we design and fabricate highly porous graphene foam from exfoliated GO paper by using candle flame treatment. The ultra-high temperature of the outer flame was found to be capable of removing the oxygen-containing groups (OCGs) on GO sheets, which not only leads to the formation of highly porous structures but also dramatically improves the conductivity of the flame reduced graphene oxide (FR-GO). The hierarchically porous architecture effectively improves the surface area, which enlarges the contact area between the electrode and the electrolyte. Therefore, the mass transfer has been significantly promoted. It needs to point out that our approach is simple, low-cost, chemical-free and environmental benign for the preparation of porous graphene anodes towards high energy density rechargeable commercial energy storage devices. We also propose that the conductive and porous graphene foam can be widely used in curvilinear electronics, catalytic, biofuel and biomimetic systems.

## Results and Discussion

**Figure 1:**
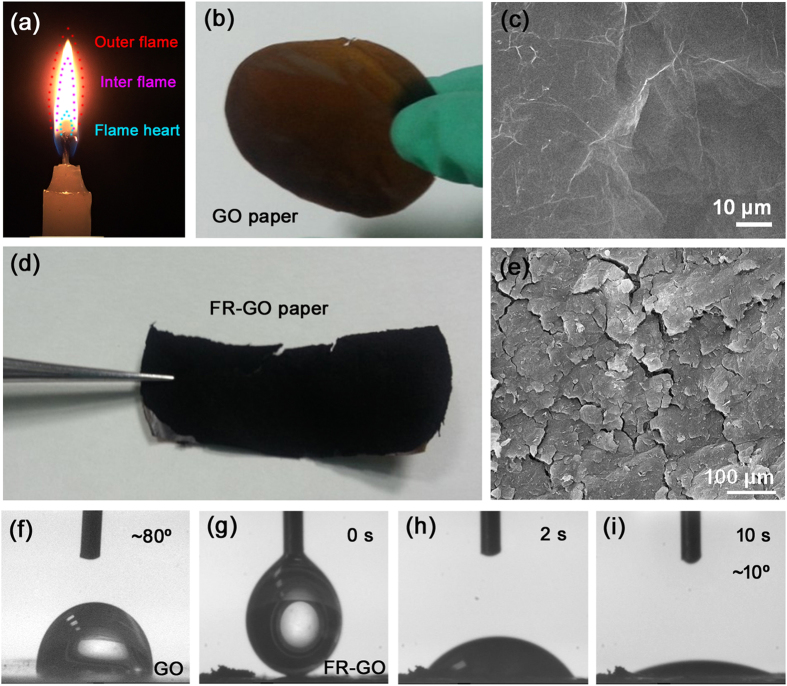
(**a**) Schematic illustration of the different regions of a candle flame, the photograph was taken by Hao-Bo Jiang; (**b**) Photograph of as prepared GO paper; (**c**) typical SEM image of the surface of the GO paper; (**d**) photograph of FR-GO prepared by candle flame treatment of GO paper; (**e**) SEM image of FR-GO sample; (**f**) Contact angle of pristine GO paper; (**g**–**i**) Contact angles of FR-GO measured at different time.

Notably, the OCGs on the surface of GO sheets would influence the interaction between lithium ions and graphene^27^. It is well known that the reaction between lithium ions and carbonyl/carboxylic acid functional groups is occurred at ~3 V vs Li/Li^+^^28^, which allows fast pseudo capacitive surface reaction (Faradaic redox reaction) of LIBs. So a reduction treatment of GO is a very important path to remove the OCGs and achieve its high conductivity. To realize this aim, we developed a smart as well as a facile strategy, flame treatment, to reduce GO papers towards energy storage applications. Briefly, we exposed the GO paper to a candle flame to trigger the drastic reduction of GO. The candle flame consists three parts: the outer flame, inner flame and flame heart^29^. Fig. 1a schematically shows the distribution of these three regions in a candle flame. Generally, the temperature of the outer flame is much higher (~1400 °C) than that of the other two parts (~650 °C for inner flame and 250 °C for flame heart, respectively)^29^. In this case, the outer flame treatment of GO could effectively reduce GO and generate hierarchically porous structures, revealing great potential for the production of graphene anodes towards energy storage applications^30^. Our GO paper was obtained by suction filtration of GO aqueous solution prepared by chemical oxidation of purified natural graphite using Hummers method. As shown in Fig. 1b, the as prepared GO paper is flat, smooth, and yellowish brown in colour. Scanning electron microscope (SEM) image shows the details of its surface topography (Fig. 1c); there exist some wrinkles and folding on the smooth surface of GO paper due to irregular stacking of GO sheets during filtration. The flame treatment of GO was performed in an ambient condition, so the treatment time should be very short, of which it is generally within a second because long-time treatment would completely combust the GO paper. The reaction time of the flame triggered thermal reduction is very short; as soon as the GO paper approaches the burning flame, the GO paper changed from yellow brown to black (Fig. 1d), the obvious colour change indicates the removal of OCGs, which is a very common phenomenon in GO reduction. SEM image of the flame reduced GO (FR-GO, Fig. 1e) shows that the neat and continuous GO paper turned into chapped film, abundant voids and cracks could be observed all over the sample. The formation of such morphology could be attributed to the thermal effect induced rapid removal of OCGs.

To evaluate the surface wettability change before and after the flame treatments, static water contact angle (CA) of the two samples was measured. As shown in Fig. 1f, the water CA of the pristine GO paper was about 80°, it is slightly hydrophilic due to the presence of OCGs. However, when the GO paper was reduced by flame treatment, the water droplet could be adsorbed by the FR-GO sample. As shown in Fig. 1g–i, within 10 seconds, the water CA of the FR-GO sample decreased to ~10°. Generally, the surface wettability of a solid film is governed by both surface chemical composition and surface roughness. By comparison of the SEM of GO and FR-GO, it could be found that the surface roughness increased due to the formation of voids and cracks. Moreover, the reduction of GO would lower its surface energy without doubt. In principle, both of the two effects would increase the surface hydrophobicity, giving rise to an increased water CA. In this case, the significantly decreased water contact angle could be attributed to the porous structure, which is of benefit to water adsorption due to the capillary action. It is worthy pointing out that such hydrophilic porous structure is very important for its application as anodes, since the interconnected porous structure would facilitate the transfer of lithium ions, and thus promote the intercalation kinetics^31^.

**Figure 2:**
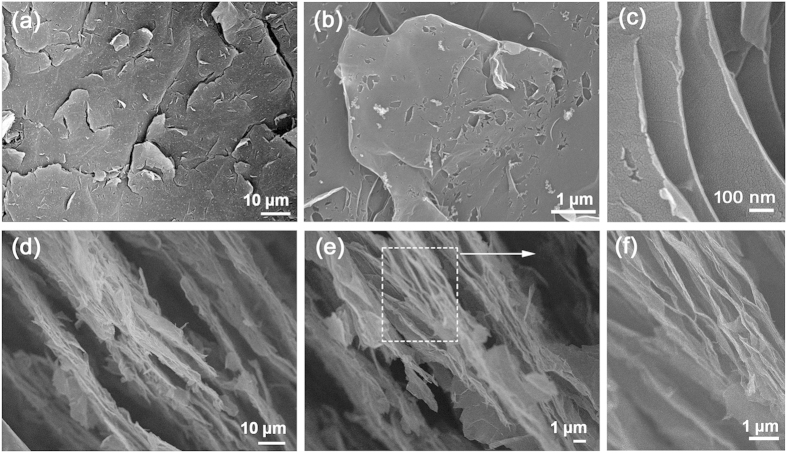
SEM images of FR-GO sample. (**a**–**c**) surface images of FR-GO; (**d**–**f**) profile of FR-GO.

Taking advantage of the high temperature of the outer flame, the GO paper has been reduced in a drastic manner. As soon as the GO approaches the outer flame, the intense thermal effect would trigger the rapid removal of OCGs, leading to the formation of a highly porous structure due to the drastic expansion. Figure 2 shows the SEM images of the resultant FR-GO samples. Notably, there are many cracks and open pores that randomly distributed on the surface of FR-GO. The formation of such porous structure could be attributed to the flame treatment, during which the sudden spurt of carbon species, such as CO, and CO_2_, would destroy the GO sheets and generate more defects. Additionally, the emission of carbon species also leads to significant expansion along the lateral direction. It could be clearly observed from the edge of the open pore that the FR-GO sheets curl up, which significantly increases the interlayer spacing. To get further insight into the porous structure along the lateral direction, we further examine the profile structure of the FR-GO sample by SEM. As shown in Fig. 2d–f, unlike the compact GO paper, the FR-GO sample expanded significantly along the lateral direction due to the degassing process. Section view of the SEM images shows obvious gaps between different layers. In addition to the large gaps, the interlayer spacing is also enlarged. Well-exfoliated porous structure could be observed all over the sample section. It is worthy pointing out that the formation of such hierarchical structures with abundant open pores would facilitate the electrolyte accessibility to the electrode surfaces, and offers an opportunity to optimize their performance in LIBs.

**Figure 3:**
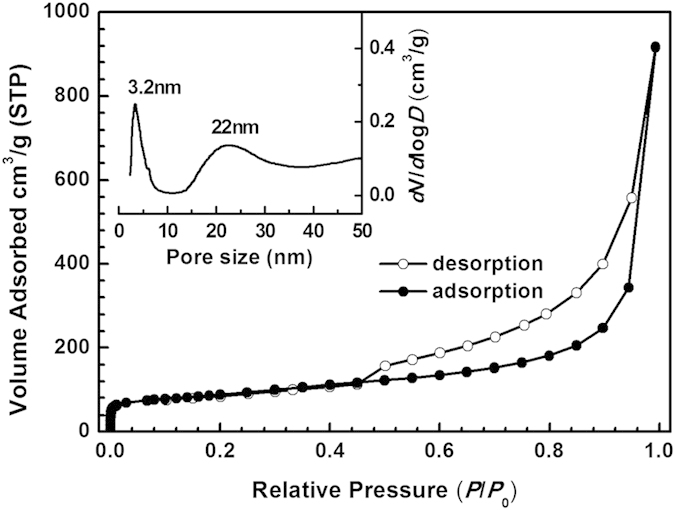
(**a**) Nitrogen adsorption/desorption isotherms of FR-GO; The inset is pore size distribution of FR-GO.

The hierarchically porous structure of FR-GO has also been proved by nitrogen adsorption experiments. Figure 3 shows the nitrogen adsorption/desorption isotherms of FR-GO sample. Notably, abrupt nitrogen uptakes are observed at low P/P_0_ region and in the range of 0.4–1.0, respectively, indicating the presence of mesopores of different pore sizes. Pore size distribution (inset of Fig. 3) confirm the hierarchical structure, mesopores with an average pore size of ~3.2 nm and 22 nm could be observed from the curve. In addition, these samples show high BET surface areas (310 m^2^/g) and large pore volumes (1.4 cm^3^/g). These results show that the FR-GO sample is highly porous, revealing great potential as anode materials for LIBs. For comparison, the GO sample shows a relative low BET surface area of 31 m^2^/g (Fig. S2).

**Figure 4:**
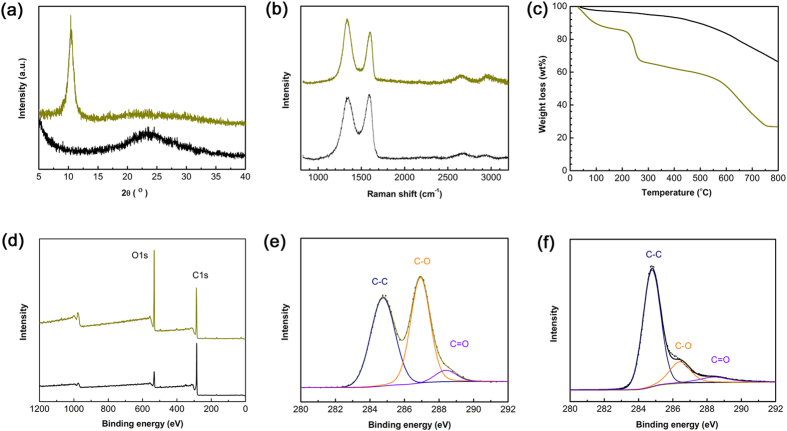
(**a**) XRD patterns of GO and FR-GO; (**b**) Raman spectra of GO and FR-GO; (**c**) DTA-TG curves of GO and FR-GO; (**d**) Survey XPS spectra of GO and FR-GO; (**e**,**f**) C1s XPS spectra of GO and FR-GO, respectively.

In order to make clear the structural and the chemical composition change before and after flame treatment, combined techniques including X-ray Diffraction (XRD), Raman spectra, TG curve and X-ray photoelectron spectroscopy (XPS) have been implemented to characterize the FR-GO sample. Figure 4a shows the XRD patterns of GO and FR-GO samples. The diffraction peak at 2θ = 10.3° with respect to GO suggested the ordered layer structure of GO sheets, a *d*-spacing of 8.6 Å could be evaluated from the pattern. However, after flame treatment, the diffraction peak disappeared, which indicates the ordered layer structure was destroyed. On the contrary, a broad diffraction peak at 2θ = 23.5° could be observed, indicating the irregular stacking of the FR-GO sheets. These results are in good agreement with the SEM images (Fig. 2).

It is well known that Raman spectroscopy has been widely used for characterizing carbon materials. As shown in Fig. 4b, the Raman spectrum of GO sample displays two peaks at 1340 cm^−1^ and 1600 cm^−1^, which correspond to *D* and *G* band, respectively. After flame treatment, the peak intensity changed slightly. The *G* band is attributed to an E_2g_ mode of graphite associated with the vibration of sp^2^ bonded carbon atoms, whereas the *D* band peak is related to the oscillations of carbon atoms with dangling bonds in plane terminations of disordered graphite. After the flame treatment, the intensity ratio of *D* band and *G* band, *I*_D_/*I*_G_, slightly decreased from 1.27 for pristine GO to 0.89 for FR-GO. The removal of oxygen defects would render a much higher content of sp^2^ carbon, represented by a significantly increased *G* band peak. The decrease of *I*_D_/*I*_G_ ratio indicates that the flame reduction of GO could effectively remove the oxygen defects and partially reconstruct the sp^2^ network of graphene.

To further evaluate the residual OCGs on FR-GO sample, thermogravimetric (TG) heating curves of GO and FR-GO have been measured in nitrogen (Fig. 4c). The weight loss below 220 °C could be attributed to desorption of water and the removal of some OCGs. In our work, pristine GO has ~20% weight loss at 220 °C, whereas the weight loss for FR-GO sample is only ~3% at the same temperature. When the temperature was further increased to 800 °C, total weight loss for GO could reach ~70%, whereas, FR-GO only shows 30% weight loss at such high temperature. According to the above analysis, it could conclude that FR-GO have been effectively reduced, the removal of OCGs directly leads to better thermal stability and less weight loss upon high temperature treatment in inert gases atmosphere.

In order to analyse the reduction degree of FR-GO sample, we measured the XPS of pristine GO and the resultant FR-GO, respectively. As shown in Fig. 3d–f, both GO and FR-GO samples have signals of carbon and oxygen. The C1s peak could be simply deconvoluted into three peaks at 284.8, 286.9 and 288.8 eV, which could be attributed to C–C (nonoxygenated ring carbon), C–O (hydroxyl and epoxy carbon), and C=O (carbonyl), respectively^32^. Notably, the content of oxygen atoms in pristine GO is as high as 33.5%, and after flame treatment, the C–O and C=O peaks decreased significantly (Fig. 3f), indicating the removal of OCGs on the GO sheets. According to the XPS results, the oxygen content of the FR-GO film decreased to 13.4%. The significantly reduced oxygen contents indicate that GO sheets have been effectively reduced after the flame treatment; it becomes conductive, and could connect the circuit and light up a LED bubble (Fig. S1). Additionally, the reduction is controllable. We stochastically choose five bathes of samples for XPS analysis, they almost show the same C1s peaks, indicting the consistent reduction degree. (Fig. S3)

**Figure 5:**
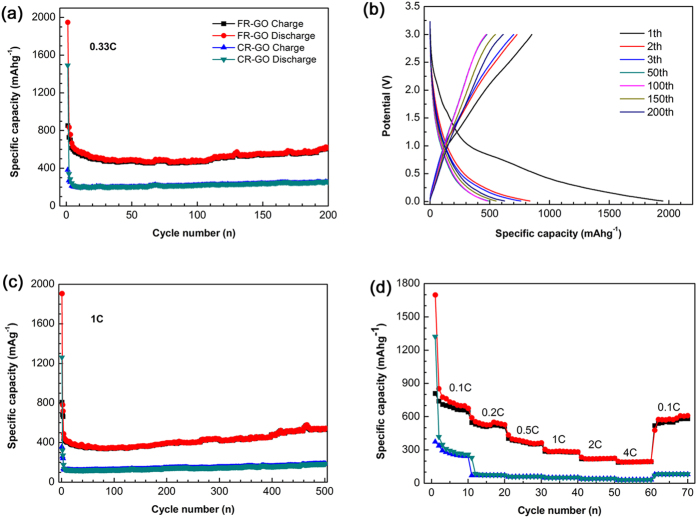
Electrochemical performance of FR-GO and CR-GO between 0 and 3 V vs. Li+/Li. (**a**) Cycling performance of FR-GO at the current densities of 0.33C and 1C; (**b**) Galvanostatic charge–discharge capacity and voltage profiles of FR-GO at 1st, 2nd , 3rd, 50th, 100th, 150th and 200th at a current density of 0.33C; (**c**) Cycling performance of CR-GO at the current densities of 0.33C and 1C; (**d**) Rating performance of FR-GO and CR-GO at various rates (0.1, 0.2, 0.5, 1, 2, 4C).

The electrochemical performance of FR-GO and chemical reduction graphene oxide (CR-GO) is shown in Fig. 5. It’s interesting to observe that the delivered capacity of the FR-GO can reach 624 mAh/g at 0.33 C (1C = 372 mA/g), which is two times than that of CR-GO (252 mAh/g) as revealed in Fig. 5a. Figure 5b displays the voltage profiles of FR-GO at 1^st^, 2^nd^, 3^rd^, 50^th^, 100^th^, 150^th^ and 200^th^ cycle at 0.33C. In the first cycle, a long voltage region below 0.5 V (*vs.* Li/Li^+^) is observed, which is mainly due to the lithium-ions intercalation into the graphene layers^33^,^34^. After the first cycle, no obvious change in the lithiation/delithiation process can be observed, indicating stable cycling performance, of which it is also consistent with the observation in Fig. 5a. Furthermore, the FR-GO and CR-GO were also tested under 1C as demonstrated in Fig. 5c. For FR-GO, a specific capacity of 541 mAh/g can be achieved after 500 cycles, which is still much higher than the value of CR-GO (186 mAh/g). Note that the delivered specific capacity keeps increasing with the cycle number increase, which is also widely observed in carbon based electrodes^35^. Herein, it needs to point out that the first Coulombic Efficiency (CE) is a little bit low, which should be assigned to the adsorption of Li^+^ on functional groups and its consumption of large amount of Li^+^ due to the formation of the SEI layer in the first cycle. It was suggested that the CE can be increased dramatically by doping graphene oxide into sucrose to reduce its specific surface area or further enlarge the interlayer lattice distance of FR-GO^36^,^37^. To study the rate performance of the FR-GO, the galvanostatic measurements were carried out at various rates from 0.1C to 4C. An excellent rate capability is revealed. The discharge capacities are 676, 529, 365, 283, 225, and 196 mAh/g at 0.1, 0.2, 0.5, 1, 2, and 4 C, respectively. It should be noted that even after sixty cycles under different current densities, it can be successfully go back as it recovers to a capacity of 610 mAh/g when the current density was changed back to 0.1C (Fig. 5d). For comparison, the rating performances of CR-GO were also carried out at 0.1, 0.2, 0.5, 1, 2, and 4 C. It can be clearly seen that it delivers rather low capacities, which is less than 100 mAh/g after the rate of 0.5 C.

The excellent performance of the as-synthesized FR-GO could be assigned to the following reasons. First, the highly porous architecture (310 m^2^/g) of FR-GO may greatly reduce the transport lengths of Li^+^, by which Li^+^ could be electrochemically adsorbed on both sides of graphene sheets^38^,^39^. Second, the self-assembled high conductive 3-D network allows the fast and continuous transport of electrons in the electrode as well as provides continuous paths, which benefits the movement of electrons over the entire electrode to achieve a high rate capability^39^,^40^. Third, the edge effect of FR-GO could enhance lithium storage because the binding energy of Li atoms on graphene sheets is superior than other carbon materials, definitely of which it depends on the morphology of edges, which have been confirmed in some related theoretical works^35^,^41^. Finally, in addition to the surface and interface effect of graphene sheets, the defects in the as-synthesized FR-GO, such as edges and vacancies, may provide more lithium storage sites, which contributes to the high reversible capacity^42^.

## Conclusion

In conclusion, we successfully developed a simple, convenient, low cost manufacturing method to produce hierarchically porous graphene materials. Flame treatments of GO films gave rise to the formation of hierarchical porosity, including microscale crack, nanoscale aperture on the graphene surface and expanded interlayer spacing, due to the high temperature annealing induced drastic degassing. The reduction effect was confirmed by both TG analysis and XPS spectra. The formation of highly porous structures and the reduction of GO make the flame treatment of GO a cost-effective strategy for preparing graphene based anode materials, since the open pores would enable facile access of lithium ions to the underlying graphene sheets and facilitate efficient intercalation kinetics significantly. It is expected that this smart method would open up a new way to prepare graphene-based materials and have broad applications in Lithium-ion battery, supercapacitor and other new fashioned electrochemical energy storage devices.

## Methods

GO was prepared from natural graphite powder using the Hummer’s method^43^. The GO paper was prepared by vacuum filtration of the GO aqueous solution through a membrane filter (0.22 μm in pore size), followed by air drying at room temperature. Finally, the GO paper was peeled off for further use. The GO paper was reduced by flame treatment, in a typical experiment, we put GO paper close to the outer flame of the candle (local temperature of the outer flame is estimated to be ~1400 °C)^44^,^45^ and the treatment time was very short (within 1 second) to avoid the burning of the GO paper.

Electrochemical measurements of the FR-GO were evaluated with LIR2032 Coin-type half-cells assembled in an argon filled glove box (MB200MOD). The working electrodes were fabricated by a mixture of 10 wt% acetylene black, 70 wt% active materials, and 20 wt% polyvinylidene difluoride (PVDF) binder with N-methyl-2-pyrrolidone (NMP) solution. Following that, the resultant slurry was uniformly coated on a copper foil with a plastic blade, of which it was put in a vacuum oven at 100 °C overnight. A Celgard 2400 microporous polypropylene membrane was applied as the separator, 1.0 mol L^−1^ of LiPF_6_ solution in mixture with ethylene carbonate (EC)–dimethyl carbonate (DMC)–diethyl carbonate (DEC) (1:1:1, in V) was utilized as the electrolyte and Li foil was used as the counter electrode.

The static water contact angle (CA) measurements were made using the Contact Angle System OCA 20 (DataPhysics Instruments GmbH, Germany) at ambient temperature. The CAs were measured by the sessile-drop method with a water droplet of 5 μL. SEM images were obtained by using a field-emission scanning electron microscope (JSM-7500F, JEOL, Japan). X-ray photoelectron spectroscopy (XPS) was performed using an ESCALAB 250spectrometer. Powder X-ray diffraction (XRD) patterns were collected on a Rigaku D/MAX 2550 diffractometer with Cu Kα radiation (λ = 1.5418 Å). Raman spectrometer equipped with a liquid-nitrogen-cooled argon ion laser at 514.5 nm (Spectra-Physics Stabilite 2017) as the excitation source; the laser power used was about 10 mW at the samples with an average spot size of 1 μm in diameter. Thermogravimetric (TG) curves were carried out on a NETZSCH STA 449C with a heating rate of 20 °C min^-1^ from room temperature to 800 °C, Ar has been used as inert gas.

## Additional Information

**How to cite this article**: Jiang, H.-B. *et al.* Flame treatment of graphene oxides: cost-effective production of nanoporous graphene electrode for Lithium-ion batteries. *Sci. Rep.* **5**, 17522; doi: 10.1038/srep17522 (2015).

## Supplementary Material

Supplementary Information
