# Supplementary material for: Flame treatment of graphene oxides: cost-effective production of nanoporous graphene electrode for Lithium-ion batteries
Source: Sci Rep. 2015 Dec 10;5:17522. doi: 10.1038/srep17522 (PMC4674706; doi:10.1038/srep17522)
Supplement: Supplementary Information [file srep17522-s1.doc]

**Supporting Information for**

Flame treatment of graphene oxides: cost-effective production of nanoporous graphene electrode for Lithium-ion batteries

Hao-Bo Jiang, Yong-Lai Zhang,* Yi Zhang, Yan Liu, Xiu-Yan Fu, Yu-Qing Liu, Chun-Dong Wang,* and Hong-Bo Sun*

((Optional Dedication))

H. B. Jiang, Y. L. Zhang, X. Y. Fu, Y. Q. Liu, and H. B. Sun
State Key Laboratory on Integrated Optoelectronics, College of Electronic Science and Engineering，Jilin University，2699 Qianjin Street, Changchun 130012, China
E-mail: [yonglaizhang@jlu.edu.cn](mailto:yonglaizhang@jlu.edu.cn); [hbsun@jlu.edu.cn](mailto:hbsun@jlu.edu.cn)

Prof. H. B. Sun
College of Physics, Jilin University, 119 Jiefang Road, Changchun, 130023, People’s Republic of China.

Y. Zhang
College of Chemistry and Molecular Sciences，Wuhan University，Wuhan, 430072, China

C.D. Wang
School of Optical and Electronic Information，Huazhong University of Science and Technology
Wuhan, 430072, China

Email: [apcdwang@hust.edu.cn](mailto:apcdwang@hust.edu.cn)

Y. Liu
Key Laboratory of Bionic Engineering (Ministry of Education)，Jilin University
Changchun 130022, China


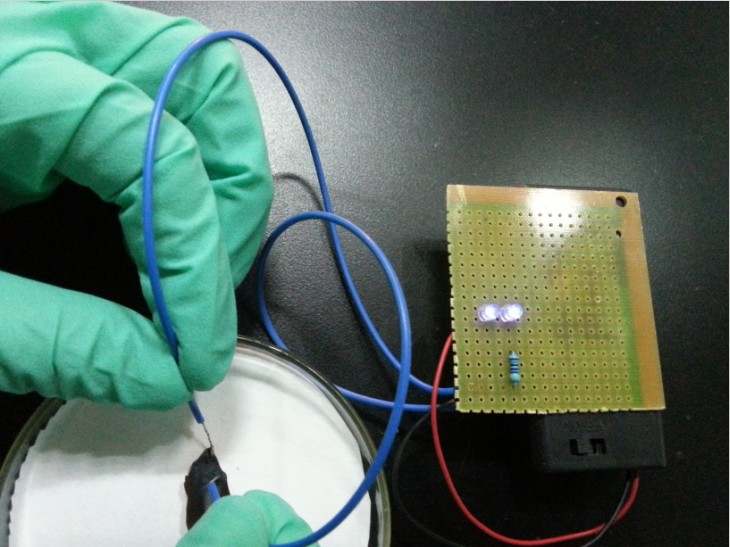


**Figure S1** FR-GO could be used for circuit connecting and light up a LED bubble, indicating its conductivity.


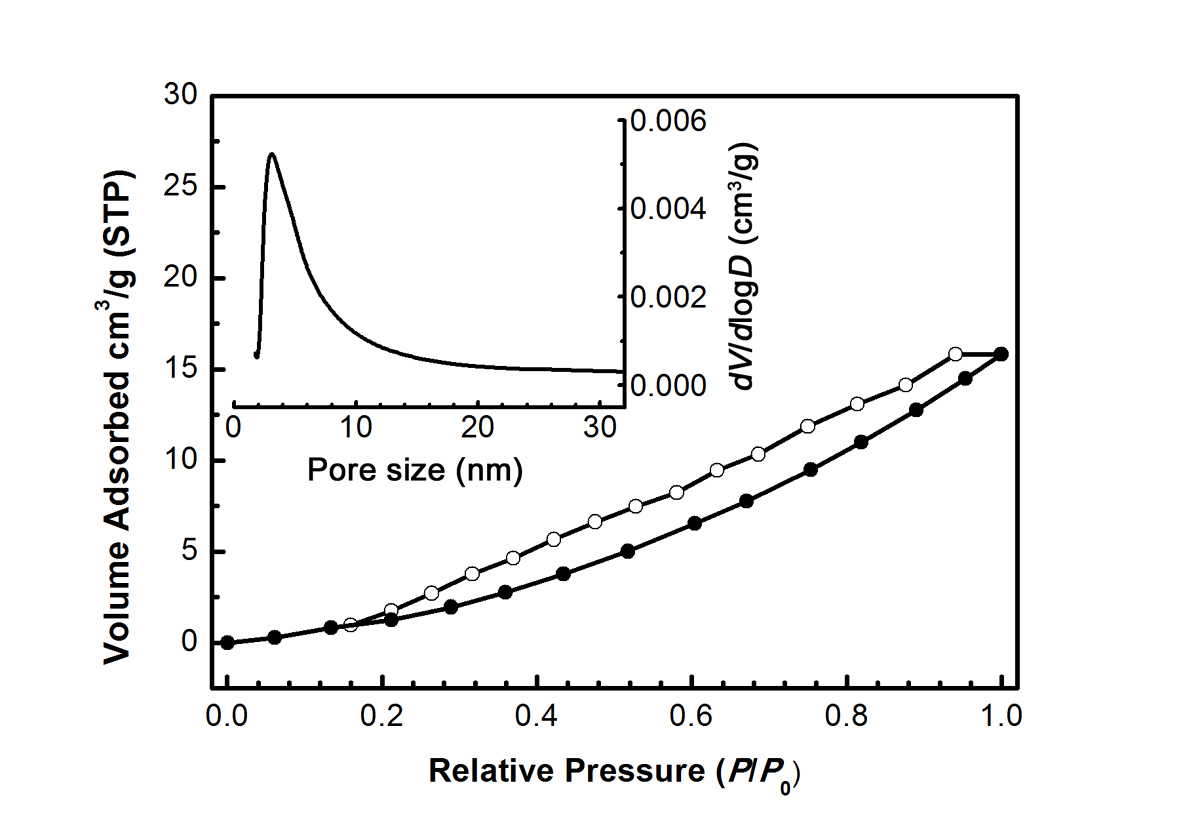


**Figure S2.** N_2_ adsorption/desorption isotherms and pore size distribution of GO.


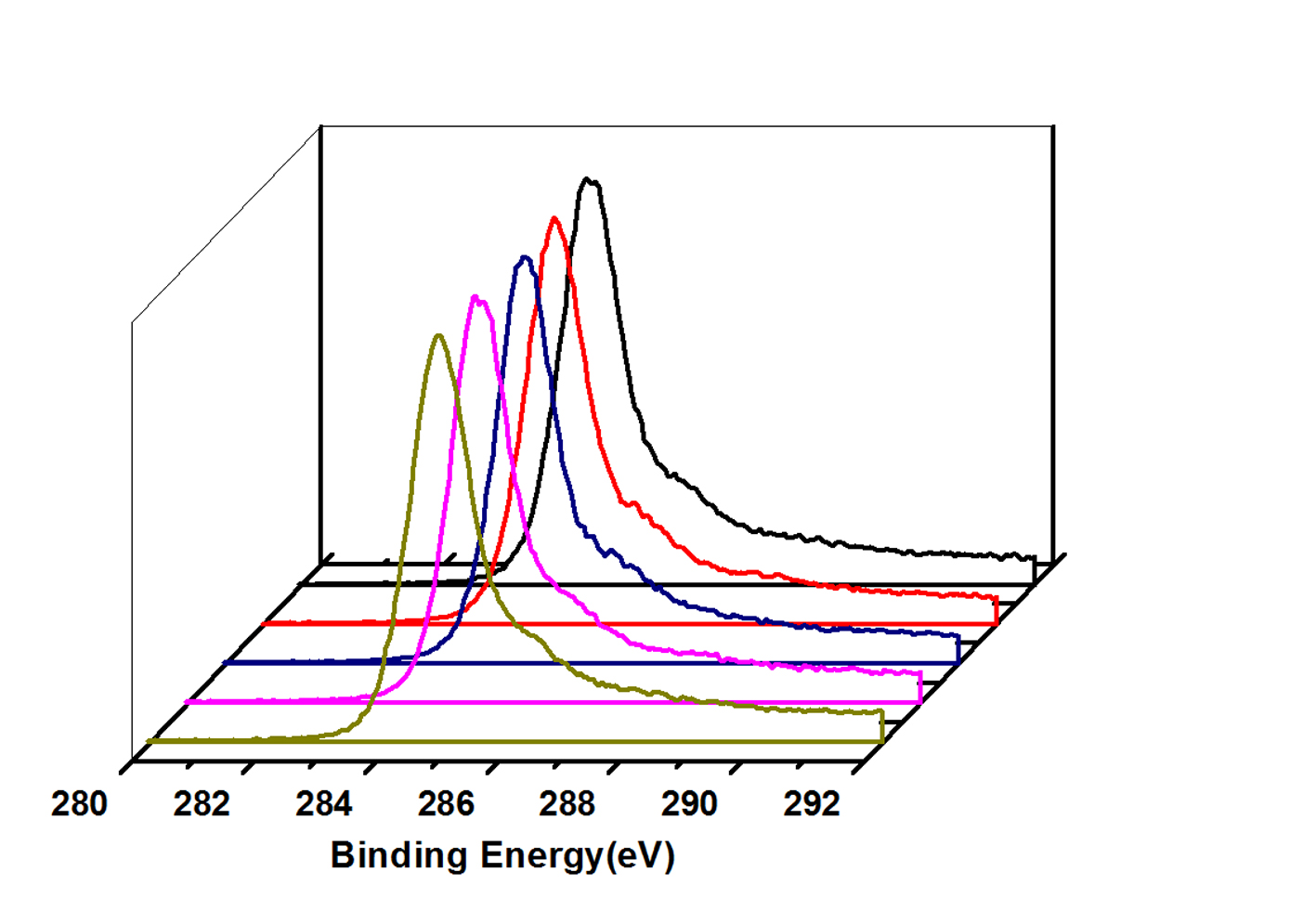


**Figure S3.** C1s spectra of FR-GO prepared from different batches.
